# The Smallest Isoform of *Metridia longa* Luciferase as a Fusion Partner for Hybrid Proteins

**DOI:** 10.3390/ijms21144971

**Published:** 2020-07-14

**Authors:** Marina D. Larionova, Svetlana V. Markova, Nina V. Tikunova, Eugene S. Vysotski

**Affiliations:** 1 Photobiology Laboratory, Institute of Biophysics SB RAS, Federal Research Center “Krasnoyarsk Science Center SB RAS”, 660036 Krasnoyarsk, Russia; larionova.marina@inbox.ru (M.D.L.); smarkova@mail.ru (S.V.M.); 2 School of Fundamental Biology and Biotechnology, Siberian Federal University, 660041 Krasnoyarsk, Russia; 3 Institute of Chemical Biology and Fundamental Medicine, Siberian Branch, Russian Academy of Sciences, 630090 Novosibirsk, Russia; tikunova@niboch.nsc.ru

**Keywords:** bioluminescence, coelenterazine, copepod luciferase, single-chain antibody, immunoassay, tick-borne encephalitis virus

## Abstract

Bioluminescent proteins are widely used as reporter molecules in various in vitro and in vivo assays. The smallest isoform of Metridia luciferase (MLuc7) is a highly active, naturally secreted enzyme which, along with other luciferase isoforms, is responsible for the bright bioluminescence of marine copepod *Metridia longa*. In this study, we report the construction of two variants of a hybrid protein consisting of MLuc7 and 14D5a single-chain antibody to the surface glycoprotein E of tick-borne encephalitis virus as a model fusion partner. We demonstrate that, whereas fusion of a single-chain antibody to either N- or C-terminus of MLuc7 does not affect its bioluminescence properties, the binding site on the single-chain antibody influences its binding capacity. The affinity of 14D5a-MLuc7 hybrid protein (*K*_D_ = 36.2 nM) where the C-terminus of the single-chain antibody was fused to the N-terminus of MLuc7, appeared to be 2.5-fold higher than that of the reverse, MLuc7-14D5a (*K*_D_ = 87.6 nM). The detection limit of 14D5a-MLuc7 hybrid protein was estimated to be 45 pg of the recombinant glycoprotein E. Although the smallest isoform of *M. longa* luciferase was tested as a fusion partner only with a single-chain antibody, it is reasonable to suppose that MLuc7 can also be successfully used as a partner for genetic fusion with other proteins.

## 1. Introduction

The resolving power of modern immunoassays depends on both specificity and affinity of antibodies as well as the efficiency of reporter molecules in converting antibody-analyte complex concentration into a detectable signal. For this purpose, antibodies or analytes are frequently linked to luciferases or Ca^2+^-regulated photoproteins [1,2,3,4]. These proteins catalyze various bioluminescent reactions and are responsible for the bioluminescence of various marine and terrestrial organisms [5]. In immunoassays they can achieve a detection threshold of down to 10^−18^ mol of analyte, taking advantage of their high quantum yield of light emission and the exquisite sensitivity of modern light detectors. To achieve covalent linkage between antibody or analyte and a protein reporter, the use of cross-linking chemicals is the norm. However, this often yields a mixture of conjugated molecules with different affinities and decreases, or even annuls, the activity of signal enzyme, diminishing detection power. Bifunctional molecules constructed by genetic engineering have a number of advantages in this respect over those produced by cross-linking chemicals. These are primarily their invariable ratio of recognizing domain/reporter protein and their constant orientation relative to each other.

Firefly luciferase and Ca^2+^-regulated photoproteins aequorin and obelin are the most widely used bioluminescent reporters in in vitro assays [1,2,3]. Ca^2+^-regulated photoproteins originating from luminous marine coelenterates consist of a ~22 kDa single-chain globular apoprotein and 2-hydroperoxicoelenterazine, noncovalently bound and stabilized within the inner cavity of the apoprotein [6,7]. Photoprotein bioluminescence is triggered by attaching calcium ions to the protein’s Ca^2+^-binding sites [5,8]. These bioluminescent proteins were fused with various recognizing molecules such as pro-ZZ protein A, antibody fragments, streptavidin etc. [2]. Although these fusion proteins reveal a high sensitivity to analyte detection in a wide linear range, they nevertheless have several shortcomings. Only the N-terminus of Ca^2+^-regulated photoproteins can be fused with a recognizing molecule, which is not always feasible. Moreover, fusion can only be performed on the apoprotein and a subsequent incubation with its substrate coelenterazine is needed to obtain the functional photoprotein. However, this procedure requires the addition of reducing agents such as dithiothreitol or β-mercaptoethanol [9] the presence of which can destroy the recognizing part of the fusion protein.

Firefly luciferase is another bioluminescent protein frequently used as a signal molecule in binding assays [3]. This type of luciferases is also single-chain globular proteins but with the molecular mass of ~62 kDa [10]. These luciferases catalyze oxidative decarboxylation reaction of its specific substrate firefly D-luciferin, also involving ATP and Mg^2+^ [5]. This reaction result is the light emission with a high quantum yield of 0.48 [11]. In most cases, it achieves analyte detection over the required range [3]. The single-chain luciferase which is responsible for bioluminescence of sea pansies *Renilla reniformis* [5] and *Renilla muelleri* [12] is another well-characterized bioluminescent enzyme. The advantage of these luciferases over firefly ones is the simplicity of the light-emitting reaction requiring only coelenterazine as the reaction substrate and oxygen. However, the quantum yield of Renilla bioluminescence is only 0.055 [13]. Efforts over the past decade to increase its quantum yield has led to Renilla luciferase mutants with a 4-fold increased light output [14,15] but these mutants have not yet found wide application in binding assays.

The bright blue bioluminescence of marine copepods arising as a secretion from epidermal glands in response to different stimuli is also attributed to luciferases using coelenterazine as a reaction substrate [4]. At the beginning of the 2000s, the first copepod luciferases were cloned from the *Gaussia princeps* and *Metridia longa* species using the functional screening [16,17]. Later, the same approach was applied to isolate three additional isoforms of the *M. longa* luciferase [18,19,20]. Based on the comparison of amino acid sequences of Metridia isoforms with each other and with those of the other copepod species, these isoforms were suggested to be the products of the four groups of non-allelic paralogous genes [20]. All copepod luciferases are single-chain proteins with the molecular mass of 18.4–24.3 kDa. The luciferases comprise a natural signal peptide for secretion, variable N-terminus constituting up to one-third of the amino acid sequence length which does not significantly influence their light emitting function [21], and a C-terminal conserved region where the enzyme active center is located [4]. This conserved region is formed by two similar repeated domains of about 70 amino acid residues in length which, in turn, include 32 highly conserved amino acid sequences, each containing five conserved Cys residues [17,22]. The presence of these cysteines suggests the presence of up to 5 S-S bonds per luciferase molecule [4] which are most likely responsible for the extreme stability of these luciferases [19,23]. Noteworthy is that even though the Renilla and copepod luciferases use the same substrate and most likely utilize the same mechanism of the substrate conversion into light, these luciferases differ in size and moreover do not share any similarity in their amino acid sequences [24].

Owing to high stability, small size, and strong bioluminescence activity, copepod luciferases have rapidly gained notice as reporters in non-disruptive assays in vivo [4,25]. While the application of copepod luciferases in various in vivo assays grows from year to year, there are only a few examples of applying them in analytical assays in vitro. The Gaussia luciferase (GpLuc) genetically fused with a biotin acceptor peptide for in vivo biotinylation in *Escherichia coli* cells was tested in a DNA hybridization assay and showed a detection limit of 1 amol [26]. Similar sensitivity was attained in the binding assay involving Metridia luciferase produced in *E. coli* and chemically modified in vitro with biotin [18]. The GpLuc conjugated with antibody to interferon-γ via genetically introduced additional N-terminal tyrosine was successfully used to determine INF-γ in human serum [27]. The approach based on the construction of fusion proteins was also tested. The Gaussia luciferase was fused with a zinc transporter protein (ZnT8) which is an autoimmune target of type 1 diabetes. It was demonstrated that ZnT8 autoantibodies can be detected in patient sera with a higher sensitivity than the commercially available ELISA kit allows [28]. Another successful example is the assay of cortisol with the use of Gaussia luciferase fused to a single-chain artificial antibody which appeared to be more sensitive than any currently available cortisol immunoassay [29].

Considering their excellent bioluminescent and biochemical properties and despite a few examples of applying Gaussia and Metridia luciferases as fusion proteins in in vivo assays [30,31,32,33], the number of reports on their use as labels in binding assays is still very limited [4]. This is mainly due to the difficulty of obtaining protein in *E. coli* cells because the correctly folded copepod luciferases must contain five intramolecular disulfide bonds [4]. Notwithstanding the recently improved procedure of obtaining one of the Metridia luciferase isoforms in *E. coli* [34], these cells still do not look promising for production of copepod luciferase fusion proteins. Especially as the fusion partner is a single-chain antibody, also containing intramolecular disulfide bonds. It seems to be much easier and more efficient to produce such fusion proteins in insect cells as secreted proteins, as this promotes proper formation of intramolecular S-S bonds.

In this study, we report for the first time the construction of two variants of a hybrid protein consisting of the smallest isoform of Metridia luciferase (MLuc7) [19] as a bioluminescent reporter and a murine single-chain variable fragment mini-antibody (scFv 14D5a) to the glycoprotein E (gpE) of tick-borne encephalitis virus (TBEV) [35], being a surface envelope protein that plays a key role in virus penetration into host cells. We also describe their secreted expression in insect cells, purification from culture medium, characterization of high purity hybrid proteins, and verification in immunoassay.

## 2. Results and Discussion

### 2.1. Genetic Constructs and Bioluminescent Properties of Hybrid Proteins

**Figure 1:**
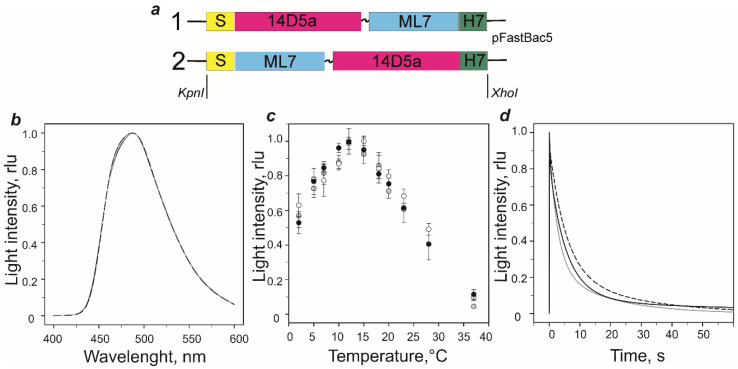
(**a**) Schematic representation of genetic constructs of hybrid proteins based on MLuc7 and 14D5a single-chain antibody for production in baculovirus expression system. S, MLuc7 secretion peptide; ML7, MLuc7 luciferase sequence without secretion peptide; 14D5a, 14D5a single-chain antibody; H7, His7-tag. (**b**–**d**) Properties of 14D5a-MLuc7 (grey lines and circles) and MLuc7-14D5a (dashed lines and open circles) hybrid proteins vs those of wild type MLuc7 (black lines or circles). (**b**) Bioluminescence spectra recorded at 23 °C. (**c**) Effect of temperature on bioluminescence activity (data represent average of 3 independent experiments for each protein). (**d**) Kinetics of bioluminescent reaction recorded at 23 °C. The measurements were performed in ML-buffer with 1:10,000 protein/substrate molar ratio. Light intensity is expressed as relative to peak values (rlu, relative light units).

**Figure A1:**
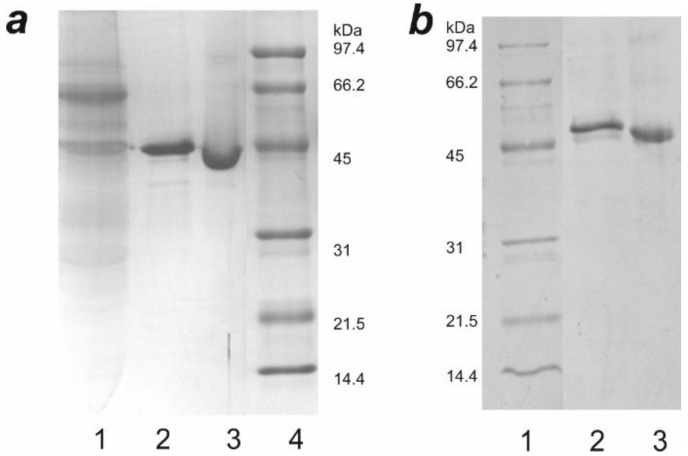
SDS-PAGE analysis of hybrid proteins’ purification from culture medium of insect cells. (**a**) Lanes correspond to 14D5a-MLuc7 samples: 1—pellet after ammonium sulfate precipitation (30–65%, *w*/*v*); 2, 3—protein samples after Ni-chromatography at denatured and semi-native conditions, respectively; 4—protein standards (Bio-Rad). (**b**) Lanes correspond to MLuc7-14D5a hybrid protein: 1—protein standards (Bio-Rad); 2, 3—samples pooled after chromatography on HisTrap column at denatured and semi-native conditions, respectively. The samples for semi-native gel electrophoresis were prepared without DTT (or β-mercaptoethanol) addition and without heating, 12.5% polyacrylamide gels were stained with Coomassie blue.

To evaluate the ability of the smallest MLuc7 luciferase to retain its functional properties as a part of hybrid protein and to determine the best options for a design of future reporter constructs, two variants of a hybrid protein (Figure 1a) consisting of the MLuc7 as a bioluminescent reporter and a murine single-chain variable fragment antibody (scFv 14D5a) against the glycoprotein E (gpE) of tick-borne encephalitis virus (TBEV) [35] were obtained. In the first variant, MLuc7 was connected as a C-terminal fusion partner of scFv 14D5a through а GSGG flexible linker to provide structural independence of the parts. In the second variant, MLuc7 and 14D5a were inversed. MLuc7 was joined as N-terminal fusion partner; the C-terminus of luciferase was fused to the N-terminus of scFv 14D5a through the same GSGG linker. For the production of these hybrid proteins and to assure the correct folding of the disulfide-rich luciferase and scFv 14D5a antibody, baculovirus expression system in Sf9 insect cells was chosen. For secretion from insect cells into the culture medium, the native signal peptide of MLuc7 was added to the N-terminus of each construct. A polyhistidine tail comprising seven histidine (H7) residues for metal affinity purification was engineered onto the C-terminus. The fusion genes for hybrid proteins were synthesized using an overlap extension PCR approach and cloned into pFastBac vector for expression in Sf9 cells. Only high purity hybrid proteins (Section 3.3) (Appendix A Figure A1) were used in the studies.

To assess the influence of genetic modification on MLuc7 activity, we studied the bioluminescent properties of the hybrid proteins. Results are summarized in Figure 1b–d and Table 1. Both proteins have peak light intensities comparable to that of wild type MLuc7. Moreover, both reveal bioluminescent spectra (λ_max_ = 487–488 nm) (Figure 1b) and temperature optima for the bioluminescent reactions (12–15 °C) (Figure 1c) identical to those of wild type MLuc7 [19]. The bioluminescence decay kinetics of both hybrid proteins, as in the case of wild type MLuc7 [36], is satisfactorily described by a two-exponential decay function only and consequently by two (“fast” (*k_1_*) and “slow” (*k_2_*)) rate constants (Table 1).

Although the decay kinetics of both hybrid proteins and wild type MLuc7 are very similar, there are some differences (Figure 1d). Fusing of the 14D5a domain to luciferase N-terminus leads to an increase of both constants whereas joining of the 14D5a domain with C-terminus results only in a rise of “slow” decay constant (Table 1). Of note is that the contribution of the “slow” component to bioluminescence kinetics increases while that of the “fast” component becomes reduced for both hybrid proteins.

The common feature of copepod luciferases is a high resistance to heat inactivation [4]. For instance, high purity MLuc7 and Gaussia luciferases produced in insect cells retain about 50% of the activity even after boiling for 1 h [20,23]. The extremely high thermostability is most likely caused by a large amount of intramolecular disulfide bonds that stabilize the structure of these luciferases [4]. We used thermal unfolding monitored by the changes in Trp fluorescence (λ_max_ = 330 nm) to evaluate whether hybrid proteins retain structural stability characteristic of copepod luciferases. The melting temperatures (T_m_) were calculated from thermal denaturation curves and were determined to be 59.2 ± 0.6 °C for both proteins. These T_m_ values are less than that determined for MLuc7 (T_m_ = 70.3 °C) but practically correspond to that of the psychrophilic isoform (MLuc2) (T_m_ = 61.4 °C) of Metridia luciferase which contains one less disulfide bond compared to MLuc7 [20].

**Figure A2:**
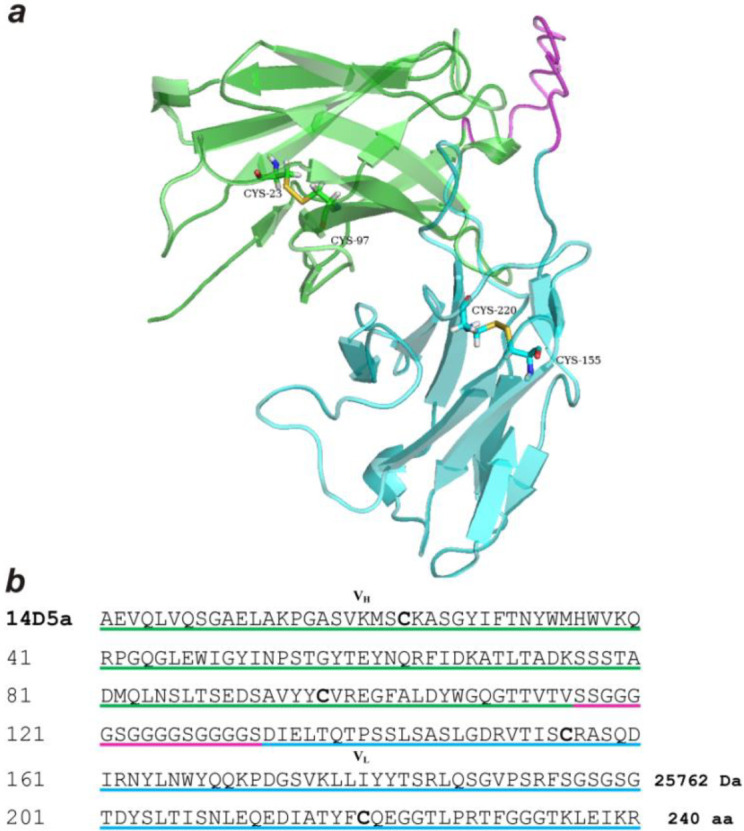
(**a**) Model for spatial structure of 14D5a single-chain antibody predicted by I-TASSER on-line service [47,48,49] showing the presence of two S-S bonds in a properly folded protein. C-score was estimated to equal 0.69 that indicates a high quality of the predicted model [47,48,49]. Cys residues forming disulfide bridges are labeled. (**b**) Amino acid sequence of 14D5a single-chain antibody. The VH and VL domains, and 17 a/a GS-bridge are underlined by green, cyan, and pink, respectively. Cys residues are shown in bold.

As we previously showed [20], MLuc7 produced in insect cells has no free thiol groups and consequently all the ten Cys residues form disulfide bonds. The hybrid proteins owing to 14D5a domain additionally comprise four cysteines which have to form S-S bonds in the correctly folded single-chain antibody (Appendix A Figure A2). Applying a standard Ellman’s test we found that both hybrid proteins have no free thiol groups under denaturing conditions (Table 1). Thus, we can reasonably assume the correct folding of both parts of hybrid proteins at their production in insect cells. The lower melting temperature of hybrid proteins as compared to that of MLuc7 can probably be attributed to either flexible linker between the domains or less structural stability of 14D5a domain, or to both factors.

### 2.2. Dissociation Constants of Hybrid Proteins

**Figure 2:**
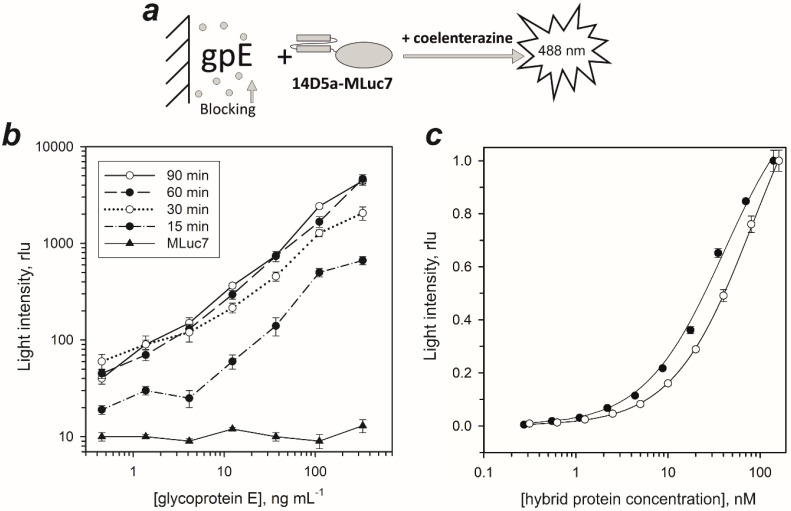
(**a**) Schematic representation of model solid-phase bioluminescent assay. (**b**) Log-log plots of the peak light intensities measured from the wells in the bioluminescent immunoassay at different incubation times of 14D5a-MLuc7 hybrid protein. MLuc7 at the concentration of 150 ng/mL was added to the wells for 1 h as a control of nonspecific adsorption caused by luciferase. The bars indicate standard deviation (*n* = 3). (**c**) Semi-log plots of dose-response curves for 14D5a-MLuc7 (●) and MLuc7-14D5a (○) hybrid proteins. L/L_sat_ is a ratio of the peak light intensity measured from a well to the peak light intensity measured from a well with a maximal light signal. Data are presented with subtracted background signals. The bars indicate standard deviation (*n* = 3). Data are presented with subtracted background signals. Nonlinear regression fitting was performed by using SigmaPlot 12.0 software. rlu, relative light units.

**Figure A3:**
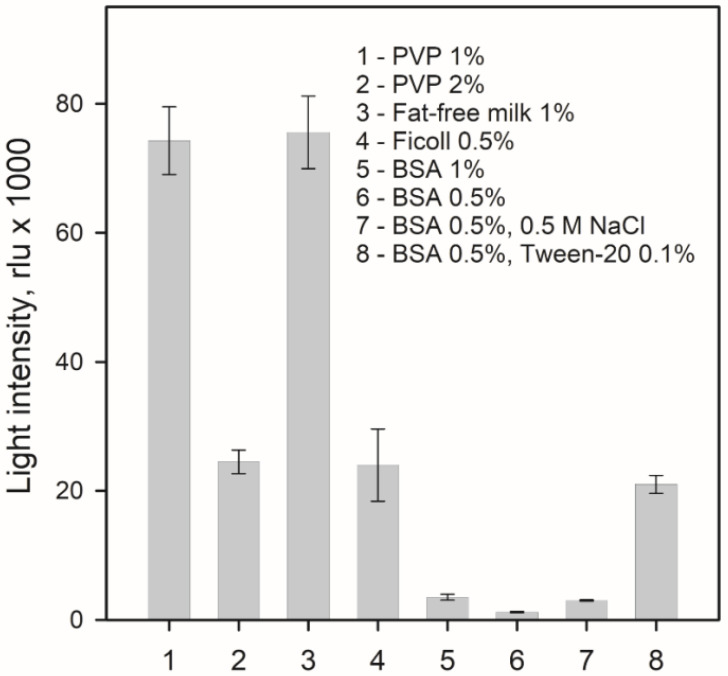
Background light signals assayed with various blocking solutions in PBS in model solid-phase bioluminescent assay (Figure 2a). Several blocking agents such as polyvinyl pyrrolidone (PVP) (Sigma-Aldrich, St. Louis, MO, USA), fat-free dry milk, Ficoll, and BSA in various concentrations with additives of NaCl and Tween-20 were tested. The bars indicate standard deviation (*n* = 3).

To address the influence of genetic modification on 14D5a, we quantified antigen-recognition of the hybrid proteins (Figure 2). Dissociation constants for the gpE-recognizing part of the hybrid proteins were determined by a non-competitive solid-phase immunoassay. Since in most cases optimal conditions can only be determined empirically, we first optimized the assay with respect to blocking solutions (Appendix A Figure A3), plate washing procedures, and incubation time of the bioluminescent probe. We found the solutions supplemented with 0.1% BSA to greatly stabilize these hybrid proteins. A solution of 0.5% BSA in PBS was most effective in blocking the non-specific adsorption of hybrid proteins, and hence the background signals. The addition of 0.07% nonionic detergent NP-40 to the washing buffer and the 5 min incubation with this buffer resulted in an additional 20-fold decrease of the background signal. The optimal incubation time to allow complete binding of hybrid proteins with gpE adsorbed on the well surface was determined to be at least 60 min (Figure 2b). We also examined whether luciferase itself tends to a non-specific interaction with the adsorbed antigen. The solution of MLuc7 (150 ng/mL) was incubated during 60 min, the wells were washed as described for hybrid proteins, and then bioluminescence was initiated by coelenterazine addition into the well (Figure 2b). Light intensity was comparable to that of the background signal without MLuc7. From this, we infer that there is negligible, if any, non-specific interaction of the luciferase part of hybrid proteins with the adsorbed gpE.

The dissociation constants for gpE-recognizing part of hybrid proteins were determined using non-competitive solid-phase immunoassay (Figure 2a) by titration of hybrid proteins on the gpE absorbed on the well surface (Figure 2c). The *K*_D_ values for 14D5a-MLuc7 and MLuc7-14D5a hybrid proteins were calculated to be 36.2 nM and 87.6 nM, respectively (Table 1). Unexpectedly, the MLuc7-14D5a hybrid protein showed ~2.5-fold less affinity than 14D5a-MLuc7. This may be accounted for by the involvement of the N-terminal part of 14D5a domain in antigen binding. Of note is that the affinity of 14D5a-MLuc7 was equal to that of the hybrid protein consisting of 14D5a domain and Renilla luciferase (14D5a-Rm7) (*K*_D_ = 37.7 nM [37]) but higher than that determined for unmodified single-chain antibody domain 14D5a (*K*_D_ = 62.5 nM [38]). In all probability, this may be attributed to the proper folding of the 14D5a domain when it is expressed in insect cells.

Since the hybrid 14D5a-MLuc7 revealed higher affinity, this very protein was used in our further experiments. We have to note that this hybrid protein can be lyophilized without any loss of bioluminescence activity and binding capacity.

### 2.3. Sandwich-Type Immunoassay of TBEV Native gpE in Tick Extracts

**Figure 3:**
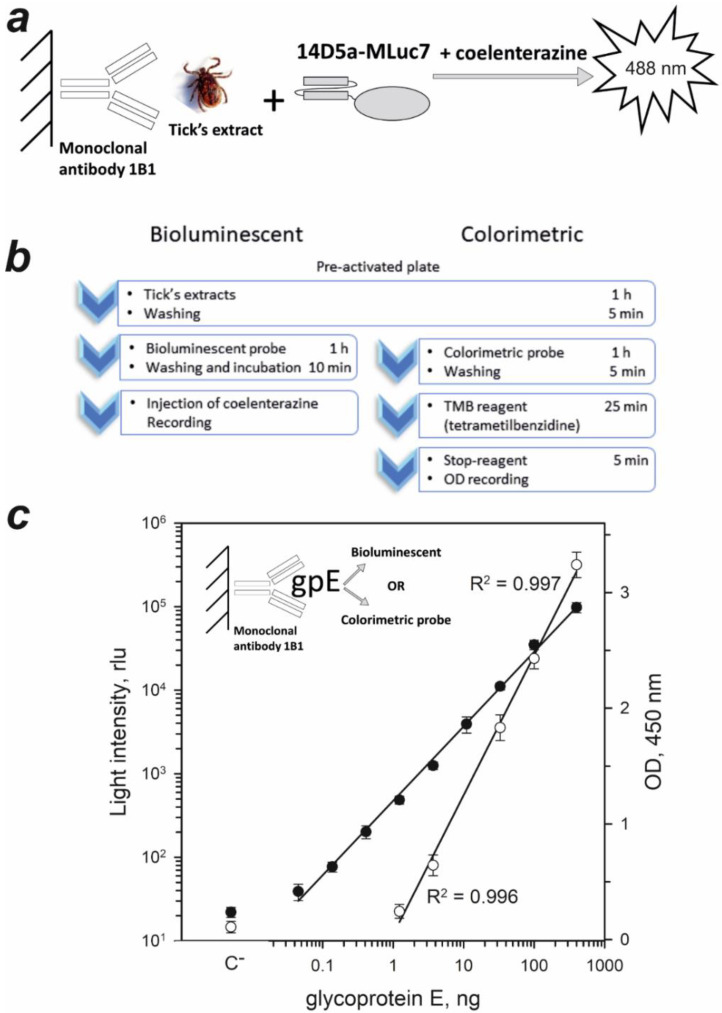
Immunoassay involving 14D5a-MLuc7 hybrid protein. (**a**) Schematic representation of the sandwich-type bioluminescent immunoassay of native tick extracts. (**b**) Steps of bioluminescent and colorimetric immunoassays of tick-borne encephalitis virus and the time required. (**c**) Sandwich-type bioluminescent (•) and colorimetric (○) assays of recombinant gpE (assay scheme is shown in the insert). The bars indicate standard deviation (*n* = 3). rlu, relative light units.

To estimate the prospects of 14D5a-MLuc7 hybrid protein as a bioluminescent probe, the sandwich-type immunoassay of TBEV native gpE in tick extracts was performed. We first determined the dose-response curve of 14D5a-MLuc7 for recombinant gpE (Figure 3a). The light signal of the 14D5a-MLuc7 hybrid protein was linear for gpE concentrations ranging from 0.045 to 400 ng (R^2^ = 0.997) (Figure 3c) and the detection limit was estimated to be 45 pg, calculated as a mean of background signals from three wells plus 2 standard deviations. For comparison, the colorimetric immunoassay with peroxidase as a label was also performed applying a commercial kit to detect TBEV native gpE (Vector-Best, Novosibirsk, Russia). According to the dose-response curve, the optical density was linearly dependent on gpE concentration in the range of 1.23–400 ng (R^2^ = 0.996) (Figure 3c). The detection limit was estimated to be 1.23 ng of gpE. Thus, sensitivity of bioluminescent assay appeared to exceed that of colorimetric assay ~30-fold. It is worth noting that gpE detection limit with 14D5a-MLuc7 hybrid protein was comparable with that determined for 14D5a-Rm7 hybrid protein (56 pg) [37], even despite the fact that bioluminescence activity was measured at room temperature which is higher than optimal for the bioluminescence activity of 14D5a-MLuc7 (Figure 1c). It is remarkable that the temperature optimum of bioluminescence reaction catalyzed by Renilla luciferase is found to be 32 °C [5] and consequently the room temperature at which the detection limit for 14D5a-Rm7 was determined is also not quite optimal for measuring the activity of this enzyme.

The bioluminescent assay of extracts from 144 ticks with the 14D5a-MLuc7 hybrid protein was carried out. In parallel, the colorimetric assay of the same extracts was also performed using a commercially available kit (Vector-Best, Russia). Among the extracts tested, only two virus-infected samples were found. The infected samples were revealed by both assays. The light signals from the wells with TBEV were at least 3 times higher than the background signals. Moreover, we did not detect any false-negative and false-positive signals for all tested tick extracts. It should be noted that the bioluminescent assay required almost half an hour less than the colorimetric assay (Figure 3b).

In addition, we estimated consumption of the 14D5a-MLuc7 hybrid protein in immunoassay. According to our calculations, 1 mg of hybrid protein can ensure more than 100,000 measurements at sandwich-type solid-phase immunoassay. Since the yield of high purity hybrid protein is 3–5 mg per liter of insect cell culture, the application of hybrid proteins using Metridia luciferase as a bioluminescent partner can be quite cost-effective.

## 3. Materials and Methods

### 3.1. Materials

Coelenterazine was purchased from NanoLight Technology, a division of Prolume Ltd. (Pinetop, AZ, USA). Its concentration was calculated by absorption at 435 nm using the ɛ_435 nm_ = 9800 cm^−1^ M^−1^ [5]. Recombinant glycoprotein E (domain III) [39] was obtained from the Institute of Chemical Biology and Fundamental Medicine, SB RAS (Novosibirsk, Russia). Extracts of ticks were supplied by the Center for Hygiene and Epidemiology of the Krasnoyarsk region (Krasnoyarsk, Russia).

### 3.2. Genetic Constructs

The own native signal peptide of MLuc7 was used for secretion of both variants of hybrid protein from insect cells. The functional domains in both hybrid proteins were fused through a GSGG flexible bridge to provide structural independence of the parts. The genetic constructs for both hybrid proteins were obtained using the overlap extension PCR approach as follows. For S-MLuc7-14D5a-H7 construct (GenBank accession number MT683623) (Figure 1a, #2), the coding sequence of *mluc7* gene with a native signal peptide was amplified by specific primers: forward primer 1 containing KpnI site (Table 2) and reverse overlap primer 2 complementary to a sequence (overlap primer 3) that encodes the end of *mluc7* fused with the beginning of the antibody *14D5a* sequence in the same reading frame through GSGG-bridge sequence. The Pfu Turbo DNA polymerase (Agilent Technologies, Santa Clara, CA, USA) was used for all reactions. Forward overlap primer 3 and reverse primer 4 were used to amplify the second part of a hybrid construct (*14D5a* sequence) using the pHEN2-14D5a plasmid, containing the VH and VL genes of the 14D5a antibody, as a template [40,41]. Then, these two fragments with overlapping ends were cleaned up using a DNA column and annealed together in equimolar amounts: the fragments in 20 μL of 4xPCR buffer were warmed up for 3 min at 94 °C and slowly cooled (20 min) to 40 °C. Then the other PCR ingredients were added to the final reaction volume of 50 μL, and a final gene splicing was carried out as follows: 5 min incubation at 72 °C, 6 cycles of PCR without primers, 6 cycles of PCR with the first forward primer 1 and a new reverse overhang primer 5. The latter was designed to introduce the C-terminal polyhistidine His7-tag followed by a TEV-specific protease site after *14D5a* or *mluc7* coding sequence and XhoI site for cloning (Figure 1a). After digestion, the synthesized fragment encoding MLuc7-14D5a-H7 hybrid protein was cloned into KpnI/XhoI sites of the modified pFastBac5 vector plasmid [20] which was produced from pFastBac1 (Life Technologies, Carlbad, CA, USA) by replacing BamHI site with KpnI site.

The S-14D5a-MLuc7-H7 hybrid protein construct (GenBank accession number MT683622) (Figure 1a, #1) in which the 14D5a part is located upstream MLuc7 luciferase in the fusion protein was similarly assembled in pFastBac5 from three partially overlapping fragments separately synthesized by PCR: (1) the sequence of signal peptide of *mluc7* gene was amplified using the pFastBac forward vector and the reverse overlap primer 6 on the obtained pFastBac5-S-MLuc7-14D5a-H7 template; (2) *14D5a* sequence was amplified with forward overlap primer 7 complementary to primer 6 and reverse overlap primer 8 using the plasmid pHEN2-14D5a as a template; (3) *mluc7-his7* sequence without signal peptide was amplified using forward overlap primer 9, complementary to primer 8, and reverse primer 5 on the pFastBac1-MLuc7-H6 template [19]. The connection of the native MLuc7 signal peptide with the antibody 14D5 did not change the position of the new signal peptide site and probability of its cleavage, as estimated by the online software SignalP 5.0 (http://www.cbs.dtu.dk/services/SignalP-5.0/index.php) [42]. Then, these partially overlapping fragments were cleaned up using a DNA column and simultaneously spliced as described above by PCR with terminal primers, pFastBac vector forward and reverse 5 primers. The final structure of the genetic constructions has been thoroughly verified by conventional Sanger sequencing of both insert strands with pFastBac vector primers performed in the Genomics Core Facility of SB RAS (ICBFM, Novosibirsk, Russia).

### 3.3. Expression and Purification of Hybrid Proteins

Generation of the recombinant bacmid DNA using DH10Bac *E. coli* cells, transfection of Sf9 cells with recombinant bacmid, the obtaining of amplified recombinant baculovirus, and virus titration were done according to manufacturer’s manual for the Bac-to-Bac Baculovirus expression system. The bioluminescence activity of hybrid proteins was used to evaluate the infection efficiency.

For secreted expression in insect cells, the Sf9 cells (Invitrogen, Carlsbad, CA, USA) cultured in suspension at 27 °C without CO_2_ using serum-free medium Sf900 II SFM (Life Technologies, Carlbad, CA, USA) with addition of antibiotic/antimycotic cocktail (Invitrogen, USA) were infected with the P2 viral stock (titer ~5 × 10^7^ infectious units per 1 mL) at a multiplicity of infection (MOI) of the 2 plaque-forming units. After three days growth with shaking at 110 rpm, the cells were harvested at 2000 *g* for 10 min at 4 °C. Then, the hybrid protein was immediately concentrated from culture medium by differential ammonium sulfate precipitation of 30–65% (*w*/*v*). The pellet was spun down (14,000 *g*, 40 min) and supernatant was additionally filtered through nitrocellulose filters (pore size 1.2 µm) to collect small insoluble particles also containing the hybrid protein. After that, both pellets were dissolved in buffer (150 mM NaCl, 20 mM imidazole, 10% glycerol, 20 mM Tris-HCl pH 7.5) supplemented with proteases inhibitors cocktail (Bio-Rad, Hercules, CA, USA) according to the manufacturer’s protocol. The solution was centrifuged (14,000 *g*, 15 min) and loaded on HisTrap column (GE Healthcare, Chicago, IL, USA) equilibrated with the same buffer. The protein was eluted by buffer containing 300 mM imidazole at 4 °C. Thereafter, the sample was concentrated with Amicon Ultra Centrifugal Filter (Merck KGaA, Darmstadt, Germany) by changing the elution buffer to the buffer containing 150 mM NaCl, 20 mM Tris-HCl pH 7.5. After chromatography, both hybrid proteins were of a high purity and monomeric according to PAGE (Appendix A Figure A1a,b), and had a molecular mass approximately corresponding to the one calculated from amino acid sequences (44.3 kDa). The yield of fusion proteins was ~3–5 mg/L of insect cell culture and corresponded to that for Metridia and Gaussia luciferases [19,20,23]. The protein concentration was determined by the BCA protein assay kit (Pierce, USA) according to the manufacturer’s manual.

For stabilizing the hybrid proteins, the 0.02% NP-40 detergent was added to the concentrated samples. The samples to be used in the bioluminescent immunoassay were additionally supplemented with 0.1% bovine serum albumin (BSA). Under these conditions, the proteins retain activity for at least six months at 4 °C. For long-term storage at −20 °C, the samples were supplemented with 50% glycerol (*v*/*v*) instead of BSA. Lyophilization of hybrid proteins from buffer (150 mM NaCl, 0.02% NP-40, 20 mM Tris-HCl pH 7.5) can also be used for long-term storage (at −20 °C for at least six months), since lyophilized samples completely restore bioluminescence activity and ability to bind target after being dissolved in water.

### 3.4. Bioluminescence Assay, Spectral Measurements, and Thermal Unfolding

Bioluminescence was measured by rapid injection of 5 µL of coelenterazine methanol solution into a luminometer cell containing a protein sample in 0.5 mL of 0.5 М NaCl, 0.015% gelatin, 50 mM Tris-HCl pH 7.5 (ML buffer). This assay buffer was used in all measurements of bioluminescence activity. The temperature of the assay tube was supported with a temperature Peltier-controlled cell holder. The luminometer was supplied with a set of neutral filters to extend the linear detection range. The specific activity of fusion proteins was estimated by the maximal luminescent signal normalized to protein concentration.

Bioluminescence decay rate constants were calculated by two-exponential fitting using the average of 3 decay curves as described elsewhere [43]. The contribution of “fast” (*k_1_*) and “slow” (*k_2_*) constants was estimated as the relative amplitude calculated from the fitted amplitudes *a* and *b* with their sum normalized to 1.

The bioluminescence and fluorescence spectra were measured with a Cary Eclipse fluorescence spectrophotometer (Agilent Technologies, Santa Clara, CA, USA) equipped with a temperature Peltier-controlled cell holder. Spectra were corrected for spectral sensitivity of the instrument. The bioluminescence was initiated by injection of coelenterazine in methanol solution (protein/coelenterazine molar ratio was ~1:1000).

Thermally induced unfolding of fusion proteins was measured by Trp fluorescence [44] (λ_ex_ = 295 nm, slit width 5 nm) at a fixed wavelength of 330 nm by gradually increasing the temperature in the protein samples from 20 to 90 °C with a heating rate of 1 °C/min. The values of thermal transition midpoint T_m_ were calculated as previously described for Metridia and Gaussia luciferases [20,23]. The measurements were performed in buffer containing 150 mM NaCl, 20 mM HEPES pH 7.5. The protein concentration was 0.1–0.15 mg/mL.

### 3.5. Determination of Dissociation Constant

The surface of microtiter strip wells with opaque flat bottom (Corning, Corning, NY, USA) was coated by recombinant gpE in PBS pH 7.5 (1 µg/mL in PBS, pH 7.5, 100 µL per well) or with 100 μL of PBS (control) by incubation at 37 °C for 1 h. Then, the solution was discarded, the wells were sequentially washed four times with a washing buffer (150 mM NaCl, 0.1% Tween, 5 mM EDTA, 100 mM K/Na phosphate buffer pH 7.0, at 23 °C), and the surface was blocked by addition of 150 µL of 0.5% BSA in PBS for 1 h at 23 °C with shaking at 350 rpm. After removing the blocking solution, the wells were washed at 23 °C four times with the same buffer and the 2-fold serial dilutions of hybrid proteins (14D5a-MLuc7 or MLuc7-14D5a in ML-buffer supplemented by 0.02% NP-40, 100 μL) with concentration in the range of 160–0.30 nM were placed into the wells. After 1 h incubation at 23 °C with shaking (350 rpm), the solution was discarded and the wells were sequentially washed 4 times at 23 °C with washing buffer supplemented by 0.07% NP-40. At the last washing step, the wells were exposed to buffer for 5 min to remove the trace amounts of hybrid protein. The bioluminescent signal was immediately measured after rapid injection of freshly prepared coelenterazine solution (100 μL per well, 1 µM in ML buffer) with a plate luminometer LB 940 Multimode Reader Mithras (Berthold, Germany). The light was measured over 5 s. The light signal maximum was used to estimate the amount of the bound hybrid protein. The light signals corrected for signal from control wells were used for plotting dose-response curves. The measurements were performed at 23 °C.

The dissociation constant was calculated according to the equation [45,46]:

(1) $$\frac{L}{L_{sat}}=\frac{\left(C+R+K_{D}\right)-\sqrt{{\left(C+R+K_{D}\right)}^{2}-4CR}}{2}$$

where *L* is the peak light intensity of the sample, *L_sat_* is the peak light intensity of the samples with saturated concentrations of hybrid protein; *R* is concentration of gpE bound to the well surface, *C* is the concentration of hybrid protein in solutions added into the wells, and *K_D_* is a dissociation constant. Parameters were calculated using SigmaPlot 12.5 software.

### 3.6. Sandwich-Type Bioluminescent Immunoassay

The surface of microtiter strip wells was coated with murine monoclonal antibody 1B1 (isotype IgG1) specific to TBEV (Biosan, Novosibirsk, Russia) (100 μL per well, 5 µg/mL in PBS pH 7.5) by incubation at 37 °C for 1 h. After the solution with antibody was removed, the wells were sequentially washed four times with washing buffer at 23 °C. (All subsequent washing procedures were always the same unless otherwise noted.) The surface of wells was blocked by 0.5% BSA in PBS (150 µL, 1 h at 23 °C with shaking at 350 rpm) and the wells were washed. Then, 100 µL of tick extracts were placed into the wells and incubated for 1 h at room temperature with shaking at 350 rpm. After removing tick extracts and washing the wells, the 100 µL solution with 14D5a-MLuc7 (70 ng/mL in ML-buffer supplemented by 0.02% NP-40) was placed into the wells and incubated at 23 °C for 1 h with shaking at 350 rpm. Then, microtiter strip wells were washed at 23 °C with the same buffer, but additionally supplemented with 0.07% NP-40. At the last washing step, the wells were exposed to buffer for 5 min to remove the trace amounts of the unbound 14D5a-MLuc7. Bioluminescence was measured as described above. The experiments were carried out in triplicate.

Commercially available immunoassay kit D1154 (Vector-Best, Novosibirsk, Russia), officially approved for medical application in the Russian Federation, which uses a horse radish peroxidase as a reporter enzyme, was applied for colorimetric determination of TBEV in the tick extracts. The assay was performed according to manufacturer’s manual.

## 4. Conclusions

In this study, we report, for the first time, the construction of hybrid proteins consisting of the smallest isoform MLuc7 of *M. longa* luciferase and the 14D5a single-chain antibody to the glycoprotein E of tick-borne encephalitis virus, their expression in insect cells and purification, their bioluminescent and biochemical properties, and binding capacity to antigen. We demonstrate that fusing of the single-chain antibody to either N- or C-terminus of luciferase does not affect its activity, temperature optimum of bioluminescence reaction, and light emission spectra, but slightly changes the kinetics of light signal. In contrast, the binding to either N- or C-terminus of the single-chain antibody does influence its binding capacity. The affinity of 14D5a-MLuc7 hybrid protein where the C-terminus of a single-chain antibody was fused with the N-terminus of MLuc7, appears to exceed 2.5-fold that of its inversed counterpart MLuc7-14D5a. Moreover, the affinity of 14D5a-MLuc7 to recombinant gpE of TBEV was even higher than that of the unmodified single-chain antibody [38]. This may be accounted for by the proper folding of 14D5a domain owing to the correct formation of disulfide bonds (Appendix A Figure A2) and consequently of the epitope recognizing region at the expression in insect cells. In addition, we show that the 14D5a-MLuc7 hybrid protein can be used not only in model immunoassay but also for the determination of TBEV native gpE in tick extracts in a sandwich-type immunoassay format. Although the smallest isoform of *M. longa* luciferase was tested as a fusion partner only with a single-chain antibody, we can reasonably assume that MLuc7 can be successfully applied as a genetic fusion partner with other proteins since modification of neither N- nor C-terminus of luciferase influences its bioluminescent activity.

Thus, although MLuc7 has some shortcomings (bioluminescence reaction optimum at temperature of 12–15 °C, difficulties in producing active luciferase in *E. coli* expression systems owing to multiple intramolecular S-S bonds) over the other bioluminescent proteins such as Renilla or firefly luciferases, MLuc7 has more advantages that outweigh its drawbacks. Primarily, these are bright bioluminescence, high thermostability, small size of enzyme that decreases probability of steric hindrances in the analysis, and possibility to modify both N- and C-terminus of luciferase. Furthermore, the efficient expression in insect cells allows obtaining a high purity hybrid protein in the amount of 3–5 mg that would be sufficient to produce immunoassay kits in enough large quantities.

## Figures and Tables

**Table 1:** Properties of hybrid proteins.

Protein	Peak Light Intensity, (rlu/mol, × 10^11^)	Biolumi- Nescence Spectrum Maximum (nm)	*k*_decay_ (s^-1^)		Free SH-Group	*K*_D_ (nM)
			*k*_1_	*k*_2_		
MLuc7	1.85 ± 0.16	488	0.20 (0.90) ^‡^ ± 0.01	0.016 (0.10) ± 0.001	0.20 ± 0.09	-
14D5a-MLuc7	1.83 ± 0.12	487	0.26 (0.73) ± 0.05	0.057 (0.27) ± 0.001	0.41 ± 0.11	36.2 ± 0.3
MLuc7-14D5a	1.80 ± 0.21	488	0.19 (0.74) ± 0.02	0.040 (0.26) ± 0.001	0.30 ± 0.07	87.6 ± 0.7

^‡^ Contribution of “fast” and “slow” components to decay kinetics.

**Table 2:** Primer sequences used for PCR at creation of genetic constructs.

No.	Primer Sequences ^†^
1	F 5′-GGCGCGGTACCATGGATATCAAATTTATTTTTG-3′
3	F 5′-GTCTTGCTGGAGATCGTGGATCCGGTGGTGCCGAGGTGCAGCTG-3′
4	R 5′-ATGATGATGACCTTGAAAGTACAAGTTCTCACGTTTGATTTCCAGC-3′
5	R 5′-TACTCGAGTCATTAGTGATGGTGATGGTGATGATGACCTTGAAAG-3′
6	R 5′-CCAGCTGCACCTCGGCAGCCTGGACCAATGCAA-3′
8	R 5′-ATTGTTTACAGTAGGGTTGCCGGATCCACGTTTGATTTCCAGC-3′

^†^ F and R, forward and reverse primers; primers 2, 7, and 9 are complementary to primers 3, 6, and 8, respectively; the cloning sites KpnI and XhoI are underlined.

## Abbreviations

MLuc7	The shortest 16.5 kDa isoform of Metridia luciferase
TBEV	Tick-borne encephalitis virus
gpE	Glycoprotein E
scFv	Single-chain variable fragment
rlu	Relative light units

## References

[1] Lewis J.C., Daunert S. (2000). Photoproteins as luminescent labels in binding assay. Fresenius J. Anal. Chem..

[2] Frank L.A., Krasitskaya V.V. (2014). Application of enzyme bioluminescence for medical diagnostics. Adv. Biochem. Eng. Biotechnol..

[3] Smirnova D.V., Ugarova N.N. (2017). Firefly luciferase-based fusion proteins and their applications in bioanalysis. Photochem. Photobiol..

[4] Markova S.V., Larionova M.D., Vysotski E.S. (2019). Shining light on the secreted luciferases of marine copepods: Current knowledge and applications. Photochem. Photobiol..

[5] Shimomura O. (2006). Bioluminescence: Chemical Principles and Methods.

[6] Head J.F., Inouye S., Teranishi K., Shimomura O. (2000). The crystal structure of the photoprotein aequorin at 2.3 Å resolution. Nature.

[7] Liu Z.J., Vysotski E.S., Deng L., Lee J., Rose J., Wang B.C. (2003). Atomic resolution structure of obelin: Soaking with calcium enhances electron density of the second oxygen atom substituted at the C2-position of coelenterazine. Biochem. Biophys. Res. Commun..

[8] Vysotski E.S., Markova S.V., Frank L.A. (2006). Calcium-regulated photoproteins of marine coelenterates. Mol. Biol..

[9] Shimomura O., Johnson F.H. (1975). Regeneration of the photoprotein aequorin. Nature.

[10] Viviani V.R. (2002). The origin, diversity, and structure function relationships of insect luciferases. Cell. Mol. Life Sci..

[11] Niwa K., Ichino Y., Ohmiya Y. (2010). Quantum yield measurements of firefly bioluminescence reactions using a commercial luminometer. Chem. Lett..

[12] Titushin M.S., Markova S.V., Frank L.A., Malikova N.P., Stepanyuk G.A., Lee J., Vysotski E.S. (2008). Coelenterazine-binding protein of *Renilla muelleri*: cDNA cloning, overexpression, and characterization as a substrate of luciferase. Photochem. Photobiol. Sci..

[13] Matthews J.C., Hori K., Cormier M.J. (1977). Purification and properties of *Renilla reniformis* luciferase. Biochemistry.

[14] Loening A.M., Fenn T.D., Wu A.M., Gambhir S.S. (2006). Consensus guided mutagenesis of Renilla luciferase yields enhanced stability and light output. Protein. Eng. Des. Sel..

[15] Stepanyuk G.A., Unch J., Malikova N.P., Markova S.V., Lee J., Vysotski E.S. (2010). Coelenterazine-v ligated to Ca^2+^-triggered coelenterazine-binding protein is a stable and efficient substrate of the red-shifted mutant of *Renilla muelleri* luciferase. Anal. Bioanal. Chem..

[16] Bryan B., Szent-Gyorgyi C. (1999). Luciferases, Fluorescent Proteins, Nucleic Acids Encoding the Luciferases and Fluorescent Proteins and the Use Thereof in Diagnostics.

[17] Markova S.V., Golz S., Frank L.A., Kalthof B., Vysotski E.S. (2004). Cloning and expression of cDNA for a luciferase from the marine copepod *Metridia longa*. A novel secreted bioluminescent reporter enzyme. J. Biol. Chem..

[18] Borisova V.V., Frank L.A., Markova S.V., Burakova L.P., Vysotski E.S. (2008). Recombinant Metridia luciferase isoforms: Expression, refolding and applicability for in vitro assay. Photochem. Photobiol. Sci..

[19] Markova S.V., Larionova M.D., Burakova L.P., Vysotski E.S. (2015). The smallest natural high-active luciferase: Cloning and characterization of novel 16.5-kDa luciferase from copepod *Metridia longa*. Biochem. Biophys. Res. Commun..

[20] Larionova M.D., Markova S.V., Vysotski E.S. (2017). The novel extremely psychrophilic luciferase from *Metridia longa*: Properties of a high-purity protein produced in insect cells. Biochem. Biophys. Res. Commun..

[21] Markova S.V., Burakova L.P., Vysotski E.S. (2012). High-active truncated luciferase of copepod *Metridia longa*. Biochem. Biophys. Res. Commun..

[22] Inouye S., Sahara Y. (2008). Identification of two catalytic domains in a luciferase secreted by the copepod *Gaussia princeps*. Biochem. Biophys. Res. Commun..

[23] Larionova M.D., Markova S.V., Vysotski E.S. (2018). Bioluminescent and structural features of native folded Gaussia luciferase. J. Photochem. Photobiol. B.

[24] Markova S.V., Vysotski E.S. (2015). Coelenterazine-dependent luciferases. Biochem. (Mosc.).

[25] Tannous B.A., Teng J. (2011). Secreted blood reporters: Insights and applications. Biotechnol. Adv..

[26] Verhaegent M., Christopoulos T.K. (2002). Recombinant gaussia luciferase. Overexpression, purification, and analytical application of a bioluminescent reporter for DNA hybridization. Anal. Chem..

[27] Moutsiopoulou A., Hunt E., Broyles D., Pereira C.A., Woodward K., Dikici E., Kaifer A., Daunert S., Deo S.K. (2017). Bioorthogonal protein conjugation: Application to the development of a highly sensitive bioluminescent immunoassay for the detection of interferon-ɣ. Bioconjug. Chem..

[28] Ustinova J., Zusinaite E., Utt M., Metsküla K., Reimand K., Huchaiah V., Merits A., Uibo R. (2014). Development of a luciferase-based system for the detection of ZnT8 autoantibodies. J. Immunol. Methods.

[29] Oyama H., Morita I., Kiguchi Y., Miyake S., Moriuchi A., Akisada T., Niwa T., Kobayashi N. (2015). Gaussia luciferase as a genetic fusion partner with antibody fragments for sensitive immunoassay monitoring of clinical biomarkers. Anal. Chem..

[30] Venisnik K.M., Olafsen T., Gambhir S.S., Wu A.M. (2007). Fusion of Gaussia luciferase to an engineered anti-carcinoembryonic antigen (CEA) antibody for in vivo optical imaging. Mol. Imaging Biol..

[31] Patel K.G., Ng P., Kuo C.C., Levy S., Levy R., Swartz J.R. (2009). Cell-free production of *Gaussia princeps* luciferase-antibody fragment bioconjugates for ex vivo detection of tumor cells. Biochem. Biophys. Res. Commun..

[32] Ng’ang´a P.N., Ebner J.K., Plessner M., Aktories K., Schmidt G. (2019). Engineering Photorhabdus luminescens toxin complex (PTC) into a recombinant injection nanomachine. Life Sci. Alliance..

[33] Gopalakrishnan R., Matta H., Choi S., Natarajan V., Prins R., Gong S., Zenunovic A., Narasappa N.N., Patel F., Prakash R. (2019). A novel luciferase-based assay for the detection of chimeric antigen receptors. Sci. Rep..

[34] Markova S.V., Larionova M.D., Gorbunova D.A., Vysotski E.S. (2017). The disulfide-rich Metridia luciferase refolded from *E. coli* inclusion bodies reveals the properties of a native folded enzyme produced in insect cells. J. Photochem. Photobiol. B.

[35] Ershova A.S., Gra O.A., Lyaschuk A.M., Grunina T.M., Tkachuk A.P., Bartov M.S., Savina D.M., Sergienko O.V., Galushkina Z.M., Gudov V.P. (2016). Recombinant domains III of tick-borne encephalitis virus envelope protein in combination with dextran and CpGs induce immune response and partial protectiveness against TBE virus infection in mice. BMC Infect. Dis..

[36] Larionova M.D., Markova S.V., Vysotski E.S. (2017). Tyr72 and Tyr80 are involved in the formation of an active site of a luciferase of copepod *Metridia longa*. Photochem. Photobiol..

[37] Burakova L.P., Kudryavtsev A.N., Stepanyuk G.A., Baykov I.K., Morozova V.V., Tikunova N.V., Dubova M.A., Lyapustin V.N., Yakimenko V.V., Frank L.A. (2015). Bioluminescent detection probe for tick-borne encephalitis virus immunoassay. Anal. Bioanal. Chem..

[38] Baykov I.K., Levanov L.N., Matveev L.E., Tikunova N.V. Development of the single-chain antibodies against tick-borne encephalitis virus. Proceedings of the conference “Genomics and proteomics in medicine”.

[39] Liapustin V.N., Karganova G.G., Sobolev S.G., Gritsun T.S. (1988). Preparative separation of the antigenic structures of the tick-borne encephalitis virus by electrophoresis in a liquid medium. Vopr. Virusol..

[40] Tsekhanovskaya N.A., Matveev L.E., Rubin S.G., Karavanov A.S., Pressman E.K. (1993). Epitope analysis of tick-borne encephalitis (TBE) complex viruses using monoclonal antibodies to envelope glycoprotein of TBE virus (persulcatus subtype). Virus Res..

[41] Baykov I.K., Matveev L.E., Matveev A.L., Tikunova N.V. (2012). Comparative analysis of variable domains of monoclonal antibodies against tick-born encephalitis virus. Sib. Med. J..

[42] Almagro Armenteros J.J., Tsirigos K.D., Sønderby C.K., Petersen T.N., Winther O., Brunak S., von Heijne G., Nielsen H. (2019). SignalP 5.0 improves signal peptide predictions using deep neural networks. Nat. Biotechnol..

[43] Eremeeva E.V., Markova S.V., Frank L.A., Visser A.J., van Berkel W.J., Vysotski E.S. (2013). Bioluminescent and spectroscopic properties of His-Trp-Tyr triad mutants of obelin and aequorin. Photochem. Photobiol. Sci..

[44] Permyakov E.A., Burstein E.A. (1984). Some aspects of studies of thermal transitions in proteins by means of their intrinsic fluorescence. Biophys. Chem..

[45] Bollen Y.J.M., Nabuurs S.M., van Berkel W.J.H., van Mierlo C.P.M. (2005). Last in, first out: The role of cofactor binding in flavodoxin folding. J. Biol. Chem..

[46] Eremeeva E.V., Markova S.V., Westphal A.H., Visser A.J., van Berkel W.J., Vysotski E.S. (2009). The intrinsic fluorescence of apo-obelin and apo-aequorin and use of its quenching to characterize coelenterazine binding. FEBS Lett..

[47] Zhang Y. (2008). I-TASSER server for protein 3D structure prediction. BMC Bioinform..

[48] Roy A., Kucukural A., Zhang Y. (2010). I-TASSER: A unified platform for automated protein structure and function prediction. Nat. Protoc..

[49] Yang J., Yan R., Roy A., Xu D., Poisson J., Zhang Y. (2015). The I-TASSER Suite: Protein structure and function prediction. Nat. Methods.

